# The use of WhatsApp group messaging in the coordination of measles supplemental immunization activity in Cross Rivers State, Nigeria, 2018

**DOI:** 10.11604/pamj.supp.2020.35.1.19216

**Published:** 2020-01-03

**Authors:** Balcha Masresha, Ogonna Nwankwo, Samuel Bawa, Thompson Igbu, Joseph Oteri, Halima Tafida, Fiona Braka

**Affiliations:** 1 WHO Regional Office for Africa, Brazzaville, Congo; 2 Department of Community Medicine, University of Calabar, Calabar, Cross Rivers State, Nigeria; 3 WHO Nigeria Country Office, Abuja, Nigeria; 4 WHO Sierra Leone Country Office, Freetown, Sierra Leone; 5 National Primary Health Care Development Agency, Abuja, Nigeria

**Keywords:** Measles elimination, Nigeria, immunization, Africa, SIAs

## Abstract

**Introduction:**

Cross Rivers State, in southern Nigeria, conducted measles Supplemental Immunization Activities (SIAs) in 2 phases from 2 -15 March, 2018. The SIAs coordination was led by the State technical coordination committee. A total of 90 supervisors from the national and subnational levels, including consultants were deployed to support the SIAs. The instant messaging service - WhatsApp was utilized to help in the communication and coordination among the State and field teams.

**Methods:**

We reviewed the chat logs from the WhatsApp group exchanges made between 28 February 2018 and 31 March 2018. Thematic content analysis was done.

**Results:**

A total of 653 WhatsApp messages were posted among the 55 group members during the study period, including text messages and media content. Eleven percent of the posts related to monitoring processes and data sharing, while posts related to vaccine logistics and waste management made up about 6% of the total. Overall coordination and deployment was covered in 6% of the posts. Forty percent of the media content showed vaccination service delivery and SIAs launching events or monitoring meetings in various areas. The coordination team used WhatsApp to send reminders to the field staff about data sharing, vaccine and waste management, as well as feedback on coverage and completeness of data sharing. The WhatsApp group discussions did not include most of the logistical and hesitancy challenges documented in the State SIAs technical report.

**Conclusion:**

We recommend focusing group discussions on instant messaging platforms so that they can be used for problem solving and sharing best practices, integrating it with other supervisory processes and tools, as well as providing feedback based on processed data from the field.

## Introduction

Nigeria has been implementing measles control strategies since 2005 and has adopted the African Regional measles elimination goal [1–3]. The strategies for attaining measles elimination include strengthening routine immunization coverage, conducting periodic Supplemental Immunization Activities (SIAs) and enhancing surveillance with lab confirmation [1]. SIAs provide an opportunity to give measles vaccine to young children that may not have received their doses in the routine immunization service. This is particularly useful in areas where routine immunization service coverage is limited. Nigeria has had annual Measles Containing Vaccine first dose (MCV1) coverage levels of less than 50% at national level [4]. With a large birth cohort and low routine immunization coverage, the number of unprotected young children accumulates rapidly posing risks of measles outbreaks. For this reason, Nigeria has been conducting periodic measles SIAs every two years in the last 12 years [2, 3]. However, SIAs play a critical role to reduce measles incidence if they can attain high coverage across all districts and if they can reach populations that are not reached through routine services. This requires early planning, the timely availability of resources, government ownership and high-level leadership, as well as intensive technical and logistical preparations, community demand generation and very good coordination [5, 6].

In 2017, The National Primary Health Care Development Agency (NPHCDA) of the government of Nigeria decided to implement the scheduled nationwide measles SIAs in a phased manner, starting with the States in northern Nigeria from October to December 2017, and covering the southern states in the first quarter of 2018, to allow for the optimal use of human and logistics resources [7]. Cross Rivers State (CRS) is a coastal state in the southern part of Nigeria. The State has 18 Local Government Areas (LGAs) further partitioned into 196 wards [8]. The projected total population of the State for the year 2018 is 4,727,230. The target population for the measles SIAs in Cross Rivers State was 714,109 children aged 9 - 59 months. The measles SIAs in CRS was carried out from 2 - 15 March, 2018. However, it was implemented in a staggered manner to maximize the available resources for the campaign. Nine LGAs were reached as part of the first phase implemented from 2nd - 7th March, 2018 while the remaining 9 LGAs were reached from 10th - 15th March, 2018 [8]. The SIAs implementation lasted for six days and two days were added to allow mop-up vaccination across all wards and LGAs.

The coordination of the SIAs in Cross Rivers State (CRS) was done by the State technical coordination committee, comprising of various officers in the State Primary Health Care Development Agency (SPHCDA), the NPHCDA, as well as partner agencies (eg., WHO, UNICEF and the African Field Epidemiology Network - AFENET). The first minute meeting of the State coordination body took place on 2nd January 2018. A total of 90 supervisors from the LGA, State and National level, as well as external consultants (recruited through the various technical support agencies) were deployed across the State to supervise, monitor and assist the implementation of the SIAs. The consultants were on board until 31st March 2018. In the weeks preceding the launch of the SIAs, and during the course of the first phase SIAs, the coordination was done from an operations room set up at the State Primary Health Care Development Agency (SPHCDA) in Calabar. The coordination of the second phase of the SIAs was done from a base in Ogoja town [8]. As part of the deliverables in the measles SIAs in Cross Rivers State, the consultants and other supervisors were requested to document their supervisory findings by filling in a questionnaire loaded on their smartphones or tablets using the Open Data Kit (ODK) software [9]. The supervisory information was supposed to be captured and submitted in real time along with time signatures and geo-coordinates [10].

At the same time, in order to address the immediate needs for regular communication and coordination of the state level officers and consultants, the State coordination team decided to set up a WhatsApp Messenger group. WhatsApp was selected because it is already widely used in Nigeria by smartphone users, is free and is considered to work well under poor network conditions. Smartphones are widely used in Nigeria. The number of smartphone users in Nigeria in 2017 was estimated to be 25 - 40 million [11]. Mobile instant messaging services offer real-time communication features. These services allow users to share text, audio, image and video messages across a range of mobile and non-mobile devices. Mobile tools have been used to facilitate supervision, program support and data sharing in the context of polio eradication activities and other disease control activities [12–14]. WhatsApp is one of many smartphone applications which are currently widely used for calls and instant messaging. The application allows many people to come together as part of a messaging group. WhatsApp group communication allows for one-to-many communication, making information generated by one member immediately available to all within the group. The role of instant messaging services like WhatsApp in health worker supervision and team building has been documented in Kenya [15]. In Mozambique, WhatsApp was used as a supplementary tool for mentoring provincial and district health teams during a campaign to distribute bed nets [16]. The authors have observed WhatsApp being used as a communication tool during immunization interventions in many countries in the African Region. In Nigeria, the national measles SIAs technical coordination body has also been using WhatsApp as a communication tool in the SIAs in the northern part of the country. However, despite the increasing use of such mobile instant messaging tools like WhatsApp, there is limited knowledge available on the ways in which these tools can be deployed to best support supplemental immunization activities. This manuscript attempts to look into the experience of using WhatsApp as a coordination tool in the context of the implementation of measles SIAs in Cross Rivers State (CRS) in Nigeria.

## Methods

The State level coordination team in Cross Rivers State decided to establish a WhatsApp group for communication purposes and it was set up on 28 February 2018. The group remains live up to April 2019. The group was administered by the CRS immunization program officer for monitoring and evaluation and a consultant supporting the State coordination of the SIAs. The members of the WhatsApp group included national and State level immunization program officers and team members, national and state level partner agency members, monitors and supervisors, as well as external consultants recruited for the SIAs by the technical agencies, all of whom were involved in the support of the SIAs operations, including logistics, communications and monitoring activities. The chat logs from the WhatsApp group exchanges were exported into MS-WORD on 16 April 2019 and reviewed. Decision was made to classify and analyze the data from 28 February 2018 (date of establishment of the group) to 31 March 2018, which was the last workday for the majority of supervisors, who were recruited as consultants. The researchers conducted the content analysis using a thematic coding system, developed after going through the posts and identifying the major programmatic areas addressed in the forum. Two researchers individually went through the chat log manually and assigned each posted message to a thematic category, based on the content of each posting. The coding assignments by the two researchers were compared and any differences in categorization was re-discussed to arrive at a consensus. Data entry and analysis was done in MS Excel. Our study did not review the supervisory information submitted using the Open Data Kit (ODK) platform.

## Results

**Figure 1:**
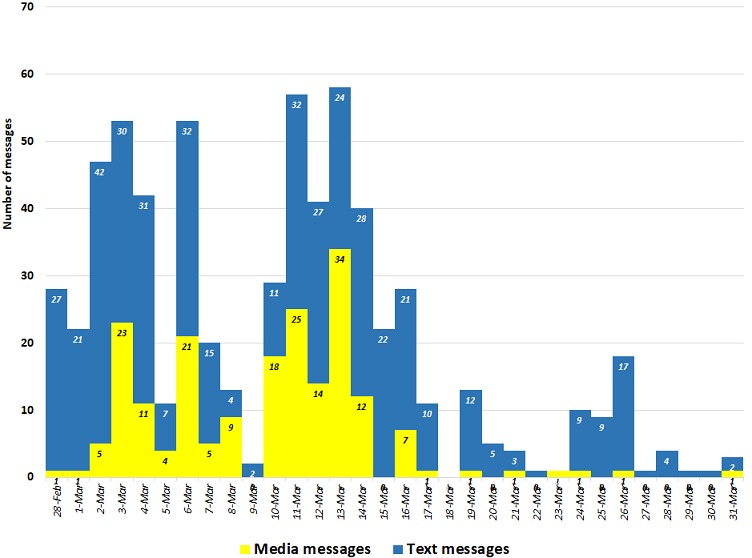
Number of text and media messages exchanged in the Cross Rivers SIAs coordination State WhatsApp group 28 February - 31 March 2018

The SIAs in Cross Rivers State were conducted between 2 - 15th March 2018. The WhatsApp group had 55 members by 31 March 2018. Members were being added into the group up to 15th March 2018. A total of 653 WhatsApp messages were exchanged among the group members during the 32 day period under study, from 28 February 2018 to 31 March 2018 (Figure 1). Five media messages could not be retrieved from the chat log. Out of the remaining 648 messages, there were 448 text messages (69%) and 200 media postings (31%), some of which included extensive captions. Out of the 200 media contents posted on the forum, 196 (98%) were images while 4 were audio or video clips. The majority of the messages (91%) shared over the period under study were exchanged from 28 February to 19th March (starting the first day the group was set up and going through to 4 days after the end of phase 2 of the SIAs in the State). The highest number of messages exchanged per day was on 13 March 2018, with 59 messages exchanged.

The thematic categorization of the group messages indicate that the largest number of messages (34%) are social exchanges among the group members including acknowledgement of prior messages, greetings, good wishes and others. There were a total of 71 posts (11%) related to monitoring processes and data sharing, while posts related to vaccine logistics and waste management made up about 6% of the total. Message postings related to technical guidance, program coordination and deployment comprised of another 6%. Posts about vaccination service delivery/vaccination post/vaccinated children as well as posts portraying best practices from the field (especially the involvement of local leaders, partners and stakeholders) each comprised about 10% of the total (Table 1). The most common type of media postings (24%) showed vaccination posts, vaccinated children and/or SIAs service delivery, while 16% of the media postings portrayed photos from SIAs launching events in various LGAs, as well as photos of observer visits from partner agencies and review meetings chaired by local authorities. The chat log includes postings containing guidance from the State/National level on vaccine logistics and waste management provided to the field staff. These included instructions to account for vaccine utilized, information about waste management and designated incineration sites. Some typical posts in this category are:

“Please State Technical Facilitators, LGA teams for both 1st and 2nd phase, be reminded of the vaccine accountability for measles vaccine: vaccine received, additional doses received, empty vials, unused vials returned (all physically counted), number of children immunized, wastage rate, remarks, etc. Documentation with the State Logistics Working Group has commenced. Thank you” Partner agency staff member.

“Please ensure filled safety boxes are moved from facility level to LGA by the Ward Focal Person. Waste management should be in the agenda for daily review meetings.” SPHCDA staff member.

“Vaccines for the second phase will arrive in the State latest today. All LGAs implementing can come for their vaccines and devices from tomorrow.” State cold chain officer.

**Table 1:** Thematic categorization of text and media messages exchanged in the Cross Rivers State WhatsApp group, 28 February 2018 - 31 March 2018

Thematic content of WhatsApp posts	Number of text messages	Number of media messages	Total number (%) of messages
Administrative content (adding group members, members leaving group, deleted messages)	67	0	6 (10%)
Acknowledgement to prior messages, greetings, good wishes, encouragements and condolences…	219	2	221 (34%)
Posts containing technical guidance, overall program coordination and staff deployment, program review exercises	30	11	41 (6%)
Posts discussing or showing vaccination service delivery / vaccination post/ vaccinated children	2	61	63 (10%)
Posts about vaccine logistics	16	1	17 (3%)
Posts on waste management	9	9	18 (3%)
Posts related to monitoring and supervisory processes	16	25	41 (6%)
Posts dealing with coverage data request, data sharing and feedback on received data/ coverage	28	2	30 (5%)
Sharing best practices from the field – involvement of local leaders, partners and other stakeholders	15	48	63 (10%)
Sharing best practices from the field - resolving problems/ reaching the unreached/ community mobilization	7	31	38 (6%)
General information from the field	18	0	18 (3%)
Posts not related to the SIAs or the group dynamics	21	10	31 (5%)
TOTAL MESSAGES	448	200	648 (100%)

Another theme that features frequently in the exchanges and discussions was monitoring and data sharing. These included reminders to the field staff to send in daily coverage statistics, supervisory data collected on the ODK platform, as well as feedback on coverage data and completeness of data sharing. The most common challenges raised by supervisors and coordinators in the field included challenges related to uploading ODK data packets and daily data transfer to the State level.

“Please remember to use your ODK implementation checklist. You are expected to upload at least 3 per day from different teams/locations/wards” Partner agency staff member.

“I have a little challenge, after filling the ODK and trying to send the final list, I noticed that (some variable fields are blank) and trying to correct it was a problem” Consultant 1 supervising implementation.

“Good morning all & welcome to day 5. The state is at 63% as at day 4. Thank God for (LGA 1, LGA 2, LGA 3 & LGA 4) for submitting day 4 data. Five other LGAs were unable to submit as at 11 pm yesterday.” Consultant 2 supervising implementation.

There were also text messages from the State level highlighting the deliverables expected from the field staff, as well as instructions on team deployment and movement and technical guidance and clarifications on technical issues.

“Each agency/national monitor on the field should remember to properly document his/her activities, achievements, etc… along with SWOT analysis following outlined report format.” Partner agency staff member.

“Sunday plan is a special plan that has list of churches with eligible children. This must include the team to visit the church, time of visit; same applicable to schools. Check your developed plans - these were included.” LGA Technical Officer.

Some of the best practices that were identified and communicated from the field included the involvement of heads of LGAs in chairing evening review meetings, resolving non-compliance on an individual level and in schools, the engagement of local authorities/partners/stakeholders in official SIAs launching events and mobilizing communities through traditional community leaders, teachers, etc.

“This (LGA name) Head of LGA´s exemplary leadership vividly translates into positive outcome as indicated by both quantitative and qualitative measles vaccination campaign results from that LGA.” Partner agency staff member.

“Attempting to convince an adamant mother to allow her 2 children to be vaccinated. Her decision of refusal was her past experience in another campaign when her child developed some AEFI and the health workers did not allay her concerns ….. And yes, we finally immunized all 2 children after the lengthy dialogue” Consultant 3 supervising implementation.

“(Name of LGA) is starting mop-up this morning. We are determined to comb all settlements and wards to ensure we have no missed children” Consultant 4 supervising implementation.

## Discussion

The information contained in the WhatsApp chat-log is highly unstructured as it contains text-messages. As the primary objective of the WhatsApp group was to share information for better coordination of the SIAs, the group communication on the forum was focused on this objective in the time period up until the end of the SIAs. On 16th March (the day after the end of the phase 2 SIAs), the first post containing unrelated subject matter was shared on the forum. One third (34%) of the posts in this WhatsApp group included civilities and encouragements exchanged between group members, which is an essential ingredient to foster team spirit as the group works towards the same goal. Overall, a total of 200 (31%) posts had media content, most of which were images showing service delivery, the conduct of meetings or SIAs launching events sent for informational purposes and occasionally captioned to showcase best practices. Around 17% of the messages were related to monitoring systems and data sharing, as well as to vaccine logistics and waste management. These messages mostly originated from the State level, and were providing reminders, specific instructions and clarifications to the field staff in order to ensure the smooth and safe conduct of the SIAs, as well as better monitoring of implementation.

The SIAs technical report for Cross Rivers State, which was compiled at the end of the exercise, identifies the following challenges; delays in the release of funds for social mobilization and SIAs logistic inputs, delayed procurement and inadequate supply of AEFI kits and cotton wool, poor road infrastructure and telecommunication network affecting staff movement and daily submission of SIAs data, late arrival of communication and mobilization materials to the State level, population non-compliance due to rumors following the monkey-pox outbreak, and the lengthy data collection tool loaded on the ODK platform for use by supervisors [8]. Most of these challenges are amendable to solutions including better coordination and alternative logistical arrangements, which require smooth communication across various levels. However, some of the challenges dealing with delayed and inadequate distribution of various materials did not feature at all in the WhatsApp group exchanges we reviewed. The problem of non-compliance and the challenges with the ODK tool were mentioned very few times in the WhatsApp group exchanges. Obviously, WhatsApp was not the only communication means that was available to the State coordination and field team. More pressing issues related to logistics as well as specific challenges are likely to have been dealt with through phone calls and face to face discussions. This may explain why some of the challenges reported in the technical report were not documented across the social media platform.

In addition, the field supervisors and consultants were expected to capture and share supervisory data using the ODK platform as a formal supervisory tool. Since data sharing is expected to be in real time, this should avail more detailed and quantified information for the State and national coordination team. We have noted that none of the posts on the WhatsApp group from the State coordination team referred to the results of analysis of ODK supervision findings at any time, nor explicitly linked guidance to the supervisory findings. WHO guidelines recommend that a provincial/ district level coordination team be in place at least 9 months before the SIAs with clear roles and tasks designated to specific members/subcommittees [6]. The State level coordination structure in CRS had its first minute meeting 8 weeks before the SIAs. The WhatsApp group was set up 3 days before the official start of the SIAs and so it was not possible to see its use in facilitating the precampaign preparedness. Countries have used WhatsApp for coordination in the context of immunization service delivery, but its use has not been analyzed or documented. In Mozambique, during a bednet distribution campaign, WhatsApp group messaging was used for coordination, and the experience among multiple groups with a total of 511 members was documented. It was noted that the use of WhatsApp was critical for implementation support to subnational level teams [16]. Other studies have documented the use of instant messaging for disease surveillance and program supervision [17, 18].

During the measles SIAs in Cross Rivers State, the administrative coverage was 103.4%. There were no severe cases of adverse events following immunization recorded during the SIAs [8]. The post-campaign coverage survey showed 88.5% coverage in the State, with 19.4% of the children having been vaccinated for the first time ever [19]. The results of this study show that instant messaging platforms are useful tools to facilitate the exchange of information and coordination among groups of people, and especially within a SIAs context. We have observed that the major part of the exchange within this WhatsApp group was more of information sharing than problem solving in nature. However, the State coordination team has used WhatsApp messaging to pass specific guidance and reminders to the supervisors in the field on key aspects of SIAs implementation, like monitoring processes and vaccine logistics.

### Limitations

Our analysis looks at only one WhatsApp group’s experience and does not represent other areas, groups or use cases. The study is descriptive and does not compare the use of WhatsApp with any other means of communication. We did not compare the findings with the outcome of the supervision done at the same time using the ODK platform. The WhatsApp group was set up three days before the SIAs implementation started and so we did not have the opportunity to see the potential uses of such communication platforms in a pre-campaign preparations setting. Moreover we looked at archived messages 1 year after the end of the SIAs, and a small proportion of the postings were lost. We cannot rule out the possibility of supervisors exercising reservations with regards to exposing logistics gaps in a group chat forum since their superiors were on the forum as well.

## Conclusion

With the increasing penetration of smartphone use and internet services, we think that instant messaging platforms like WhatsApp provide a very convenient means for one-to-many communication and can be used as supplementary tools in the coordination of public health interventions like SIAs. However, the use of such platforms can be better focused to respond to the program needs, if clear rules are set out governing the group communication; the objectives and life span of the group, as well as the inclusion and exclusion criteria are clearly mapped out from the outset. Adequate linkage needs to be made with other supervisory processes and tools (eg., integrated supervision data). Moreover, the platform can be better managed for problem solving and sharing best practices, especially if the central coordination team harvests critical information and interprets important lessons from the posts, generates program action, and provides feedback to the group. The sharing of maps, charts and other graphics related to the SIAs logistics or monitoring outputs helps the field team better visualize and capture information.

### What is known about this topic

- Instant messaging services are widely used applications;
- Coordination is an important role in the success of SIAs and smooth communication is a key part of coordination of public health interventions like SIAs.

### What this study adds

- WhatsApp messaging can serve as an important supplementary tool for better SIAs coordination;
- Careful management of a WhatsApp group communication platform along with clear protocols for use can make it into a more useful tool for SIAs coordination, problem solving and sharing best practices.

## Competing interests

The authors declare no competing interests.
